# Intraocular pressure changes under an atmospheric pressure spectrum in a multiplace hyperbaric chamber

**DOI:** 10.5935/0004-2749.2022-0085

**Published:** 2023-03-08

**Authors:** Sara Edith Moreno Mazo, Fernando Yaacov Peña, Johanna Victoria Osorio Ramírez

**Affiliations:** 1 Hospital Militar Central, Bogotá, Colombia; 2 Armada Nacional de Colombia, Hospital Naval de Cartagena, Provincia de Cartagena, Bolívar, Colombia

**Keywords:** Atmospheric pressure, Tonometry, Intraocular pressure, Ocular hypertension, Glaucoma, Military personnel, Pressão atmosférica, Tanometria, Pressão intraocular, Hipertensão ocular, Glaucoma, Militares

## Abstract

**Purpose:**

To evaluate the influence of atmospheric pressure changes on the behavior of intraocular pressure of healthy military individuals-students and instructors of the National Navy’s Diving & Rescue School at the “ARC BOLÍVAR” naval base-during a simulated immersion in the hyperbaric chamber of the Naval Hospital of Cartagena.

**Methods:**

A descriptive exploratory study was performed. The intraocular pressure was measured at different atmospheric pressures during 60-min sessions in the hyperbaric chamber while breathing compressed air. The maximum simulated depth was 60 feet. Participants were students and instructors of the Naval Base’s Diving and Rescue Department.

**Results:**

A total of 48 eyes from 24 divers were studied, of which 22 (91.7%) were male. The mean age of the participants was 30.6 (SD=5.5) years, ranging from 23 to 40. No participant had a history of glaucoma or ocular hypertension. The mean base intraocular pressure at sea level was 14 mmHg, which decreased to 13.1 mmHg (decreased by 1.2 mmHg) at 60 feet deep (p=0.0012). However, during the safety stop at 30 feet, the mean IOP kept decreasing until reaching 11.9 mmHg (p<0.001). By the end of the session, the mean intraocular pressure reached 13.1 mmHg, which is inferior and statistically significant when compared with the intraocular pressure base mean (p=0.012).

**Conclusions:**

In healthy individuals, the intraocular pressure decreases when reaching a depth of 60 feet (2.8 absolute atmosphere pressure) and it decreases even more during ascension at 30 feet. Measurements at both points were significantly different when compared with base intraocular pressure. The final intraocular pressure was lower than the baseline intraocular pressure, suggesting a residual and prolonged effect of the atmospheric pressure on intraocular pressure.

## Abstract (pt)

**Objetivo:**

Avaliar a influência das alterações da pressão atmosférica no comportamento da pressão intraocular de indivíduos militares saudáveis-alunos e instrutores da Escola de Mergulho e Resgate da Marinha Nacional na base naval “ARC BOLÍVAR”-durante uma imersão simulada na câmara hiperbárica do Hospital da Marinha de Cartagena.

**Métodos:**

Realizamos um estudo exploratório descritivo. A pressão intraocular foi medida em diferentes pressões atmosféricas durante sessões de 60 minutos na câmara hiperbárica respirando ar comprimido. A profundidade máxima simulada foi de 60 pés. Os participantes eram alunos e instrutores do Departamento de Mergulho e Resgate da Base Naval.

**Resultados:**

Quarenta e oito olhos de 24 mergulhadores foram estudados. Vinte e dois participantes (91,7%) eram do sexo masculino. A média de idade dos participantes foi de 30,6 (DP=5,5) anos, variando de 23 a 40. Nenhum participante tinha histórico de glaucoma ou hipertensão ocular. A média de base da pressão intraocular ao nível do mar foi de 14 mmHg, diminuindo para 13,1 mmHg (queda de 1,2 mmHg) a 60 pés de profundidade (p=0,0012). Entretanto, durante a parada de segurança a 30 pés, a pressão intraocular média continuou diminuindo até atingir 11,9 mmHg (p<0,001). Ao final da sessão, a pressão intraocular média atingiu 13,1 mmHg, valor inferior e estatisticamente significativo quando comparada à média de base da pressão intraocular (p=0,012).

**Conclusões:**

Em indivíduos saudáveis, a pressão intraocular diminui ao atingir uma profundidade de 60 pés (2,8 de pressão atmosférica absoluta) e diminui ainda mais durante a ascensão a 30 pés. As medidas em ambos os pontos foram significativamente diferentes quando comparadas à pressão intraocular de base. A pressão intraocular final foi menor do que a pressão intraocular de base, sugerindo um efeito residual e prolongado da pressão atmosférica sobre a pressão intraocular.

## INTRODUCTION

Glaucoma is a group of eye conditions that damage the optic nerve, generates a partial loss of the visual field (VF), and is usually linked to increased intraocular pressure (IOP). Although this increase in IOP is not a condition for glaucoma damage, it is considered a higher risk factor in the development and progression of glaucoma and, in itself, the only factor that can be modified^(1)^. Longand short-term IOP values vary during the day. Short-term IOP is affected by daily living activities that increase or lower blood osmolarity (such as eating or drinking) and as a response to sudden changes in blood pressure (BP)^(2)^. In long-term IOP, regular fluctuations that follow a circadian pattern similar to cortisol occur. Drance found that the daily IOP variation range in individuals with glaucoma is 2-3 times higher than in normal individuals^(3)^.

The eye blood flow (EBF) is determined by the ocular perfusion pressure (OPP), which drives blood toward the ocular tissues, and the resistance^®^ offered by the blood vessels to the said flow^(4)^. Since the OPP is calculated as the difference between the ophthalmic artery pressure (which corresponds to 2/3 of the mean brachial artery pressure due to the height difference between the heart and the eye) and the ocular venous pressure, which under normal conditions equals or slightly exceeds the IOP, we can relate IOP to PVO.

*Ocular blood autoregulation* is the ability of the vascular bed to modify its resistance, either through vasodilation or vasoconstriction, to maintain optimal blood flow to the tissues in all situations, compensate for changes in OPP, adapt the blood flow to the metabolic needs, and maintain the temperature of the posterior pole^(5)^. Ocular autoregulation can be triggered by multiple local or systemic, intrinsic, or extrinsic mechanisms, including neurogenic, myogenic, metabolic, and humoral factors, which are mediated by substances released by nerve endings, endothelium, or glial cells that surround the vessels or reach the eye by systemic circulation^(6)^.

The influence of external factors such as atmospheric pressure on IOP is not well known. Individuals who experience pressures >1 absolute atmosphere pressure (ATA) such as military rescue divers, go through significant changes in optic and eye physiology and occasionally experience ophthalmological disorders with various clinical manifestations. At sea level, humans are subjected to ATA. This magnitude can be expressed in other equivalent units, for instance, 760 mmHg, 33 feet of seawater (FSW), and 14.7 pounds per square inch. However, 1 ATA is the standard reference for atmospheric pressure measurement. Accordingly, when an IOP is said to be 15 mmHg, it is 15 mmHg above the external pressure; in this case, the absolute IOP at 1 ATA is 775 mmHg (760 ± 15 mmHg). Pressure measured with a tonometer is a “calculated” pressure resulting from subtracting the 1 ATA constant. When FSW is used to measure depth, ATA = depth in feet + 33^(7^-^9)^.

The effects of atmospheric pressure on the cardiopulmonary system include increased hydrostatic pressure, mainly in the chest wall, with the consequent reduction in the functional residual capacity and respiratory reserve volume, which, added to the increase in the density of air in the lungs, increases the ventilatory effort of up to 60% and causes a drop in the vital capacity of the lungs. At the cardiovascular level, peripheral blood vessels are constricted, with an increase in venous return to the thorax of approximately 800 mL, generating an increase in cardiac preload in the systolic volume and decreasing the cardiac output, with secondary arterial hypertension^(10)^.

Although a few clinical studies have addressed the influence of atmospheric pressure on ocular dynamics, a study demonstrated that the IOP of healthy individuals who are subjected to pressures above that of the sea level suffers a slight but sustained decrease because they experience greater pressure in a hyperbaric chamber and that these changes are independent of BP changes and cornea thickness^(11)^.

Oxygen (O_2_) also influences ocular perfusion because hyperoxia induces powerful vasoconstriction of the retinal vascular bed induced by the release of endothelin-1, decreasing blood flow to protect tissues from the toxicity of high O_2_ concentrations. In turn, hypoxia causes an accumulation of extracellular lactate products of retinal metabolism, which induces the release of nitrous oxide by the endothelium and subsequently vasodilation. By contrast, choroidal circulation appears to be unaffected by changes in O_2_ levels. Ersanli et al. described a significant decrease in IOP when individuals were exposed to 2.5 ATA and breathed hyperbaric oxygen, and they reported that this was caused by induced vasoconstriction due to tissue hyperoxia^(12)^.

To determine the effectt of barometric pressure on intraocular pressureto in healthy participants, i.e., students and instructors of the National Navy’s Diving and Rescue School at the “ARC Bolívar” naval base, IOP was measured at different values of atmospheric pressure, while under simulated atmospheric changes in the multiplace hyperbaric chamber of the Naval Hospital of Cartagena. In this study, individuals breathed compressed air to simulate the physiological conditions of regular immersion.

## METHODS

A descriptive study was performed to determine IOP changes at different atmospheric pressures. A convenient sample size was used, where all adult divers, either professional or in-training, from the Diving and Rescue School at the “ARC Bolívar” naval base in Cartagena were invited to participate in this study voluntarily, and they provided informed consent. Twenty-four healthy divers were exposed to different pressures, simulating different depths, in a multiplace hyperbaric chamber (type 36b special, La Spezia, Italy) at the Colombian National Navy’s Naval Hospital in Cartagena. Complete ophthalmological examination was conducted on each participant, which included visual acuity, biomicroscopy, gonioscopy, IOP measurement with applanation tonometer (three measurements, and the result was the mean of these), and macula, optic nerve, and excavation evaluation by ophthalmoscopy, white-white computerized visual campimetry (VF), complete threshold strategy (Oculus Center Field, Spak Precision software, Model Vismec IMC, serial 2556, Arlington, WA, USA), optical coherence tomography (OCT, Haag-Streit, Octopus 900, Mason, OH, USA), and pachymetry. No participant had a history of previous glaucoma or ocular hypertension. When correlating VF, OCT, IOP, iridocorneal angle, excavation, and clinical history, no participant was diagnosed with glaucoma or was suspected of having glaucoma. Two simulated immersions were performed, and divers were examined in the sitting position. The baseline IOP at sea level was 1 atm (1 ATA). The IOP in the chamber was measured with a previously calibrated Tonopen XL. IOP measurements were taken at simulated depths every 20 feet until 2.8 ATA (60 feet) were reached and during simulated ascension at 50 and 30 feet. Each stage had a 10-min waiting time between measurements. The total cycle in the chamber took 60-min. In total, seven IOP measurements were taken for each participant. No issues were encountered during the procedure. No ozone or hyperbaric oxygen was used. Instruments were calibrated, and information was filed in duplicates to avoid errors in the database. We used a Microsoft Excel database and performed statistical analyses using IBM SPSS Statistics for Mac version 22 (IBM Corp., Armonk, NY, USA). The IOP was adjusted to central pachymetry analysis using the risk progress nomogram for ocular hypertension^(13)^. To calculate the appropriate atmospheric pressure at each depth, we used the following formula: ATA = (depth + 33)/33. A descriptive analysis of the demographic and clinical variables was performed. Distribution frequencies and graphics were used for categorical variables. Central tendency, position, and dispersion or variability were used for continuous variables.

## RESULTS

**Figure 1:**
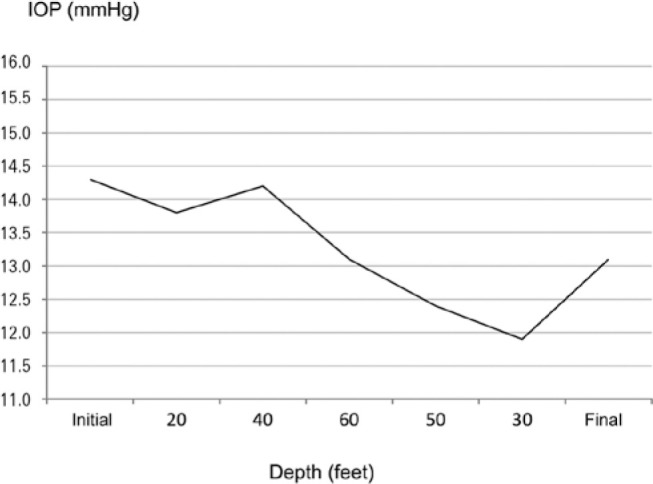
Variation of the intraocular pressure during the immersion and ascent in the multiplace hyperbaric chamber.

Forty-eight eyes from 24 divers were studied. Twenty-two participants (91.7%) were men, and the mean age of the participants was 30.6 years. The central pachymetry mean was 543.7 (SD=30.4) microns, which was used to adjust the IOP (Table 1). The mean adjusted IOP decrea-sed from sea level (14.3, SD=2.2 mmHg) until reaching 60 feet and 2.8 ATA (13.1, SD=2.6 mmHg), having a difference of -1.2 mmHg, which was statistically significant (p=0.012) (Table 2). During the safety stop at 30 feet, the mean IOP kept decreasing, and it stayed below 11.9 (SD=1.7) mmHg (p<0.001). By the end of the session, the mean IOP reached 13.1 mmHg (SD=2.6), which was inferior and statistically significant when compared with the initial mean IOP (p=0.012) (Figure 1).

**Table 1:** Characteristics of the study participants

Characteristics	Average (SD)	Range
Age (years)	30.6 (5.5)	23-40
Weight (Kg)	75.5 (8.2)	50-90
Height (m)	1.75 (0.07)	1.60-1.87
Body mass index	24.5 (2.4)	19.5-28.7
Spherical equivalent	-0.04 (0.8)	-2.1 to +1.3
Optic disk cup	0.32 (0.14)	0.0-0.54
Central pachimetry (microns)	543.7	489-624

**Table 2:** Changes in IOP according the depth and absolute pressure in the multiplace hyperbaric chamber

Time (minutes)	Depth (feet)	Absolute pressure (ATA)	Average IOP (mmHg)	Standard deviation (mmHg)	Range (mmHg)	p-value
0	0	1	14.3	3.3	6-22	-
10	20	1.61	13.8	3.2	5-20	0.095
20	40	2.21	14.2	2.6	8-20	0.810
30	60	2.82	13.1	2.4	8-18	0.012
40	50	2.52	12.4	3.2	4-19	0.000
50	30	1.91	11.9	2.7	4-17	0.000
60	0	1	13.1	2.9	6-19	0.012

ATA= atmospheric pressure; IOP= intraocular pressure.

## DISCUSSION

In this study, the average IOP decreased proportionally with the absolute atmospheric pressure in the hyperbaric chamber. This has previously been reported by other authors. Vercellin et al. compared two tonometers at different ATA conditions up to 4 bar on three days^(14)^. In the present study, we evaluated the IOP on the same day to avoid any bias regarding changes in the temperature or condition of the participants, such as previous meals, stress, and other conditions that could be interfering with the objectivity of the measurements. Gallin-Cohen et al. also used higher concentrations of oxygen and attributed the decrease in IOP to oxygen rather that the ATA pressure^(15)^. In this study, we did not change the concentration of oxygen or the hyperbaric oxygen used in the multiplace chamber. Van de Veire et al. found that the IOP decreased with a smaller variation of the atmospheric pressure, as low as 2 bars. We also observed a decrease in IOP at 2 ATA, and this effect was magnified when going up to 2.82 ATA, and the decrease in the IOP was even greater during the decompression phase^(16)^.

The continuous decrease in IOP (of 1.4 mmHg mean), reached at a maximum depth of 60 feet, which continued during decompression, stayed below the baseline throughout the immersion. This finding hints at IOP being related to the time during which the eye is subjected to environmental pressure changes. Our findings are consistent with the literature, even though the behavior of IOP is still being studied^(17)^. Other studies reported comparable results, in which partial oxygen pressure, humidity, and temperature were constant inside the chamber, but the atmospheric pressure reached a maximum of 2.5 ATA, with the participants breathing 100% oxygen^(18)^. The mean IOP drop in these studies was between 0.4 mmHg and 3 mmHg. Van de Viere reported that IOP stayed under baseline by the end of a 60-min session, similar to the results of the present study, and it was attributed to a prolonged external effect of the atmospheric pressure on IOP.

IOP measurement during simulated immersions in a hyperbaric chamber allowed the assessment of the physiological behavior of aqueous humor drainage in healthy eyes. The controlled atmospheric pressure showed that IOP decreases under these conditions. Although only a few studies are similar to the present study, the protocol used during the present study-which gives standards and expected values-might suggest, in principle, a possible benefit from hyperbaric therapy in patients with glaucoma and whose aqueous humor dynamic is altered.

The results of this study indicate that diving poses no risk regarding glaucoma development in healthy individuals because a 1 ATA increase did not lead to an increase in IOP. They also suggested that hyperbaric medicine could be a therapeutic alternative for glaucoma management because high atmospheric pressure and hyperbaric oxygen caused a decrease in IOP^(3^,^7)^.

Due to limitations of the study, we could not increase the size of the sample by examining all the students of the diving and rescue school, because some of them were involved in their own work activities.

In the future, this same study could be performed with National Air Force pilots who, during their training, are subjected not to very high pressures but to hypobaric conditions. A hypobaric chamber in Bogotá, in one of the military health facilities, could be used for this purpose.
